# Cerebral granulomatosis as a manifestation of Crohn’s disease

**DOI:** 10.1186/s12883-018-1163-8

**Published:** 2018-10-03

**Authors:** Konrad Whittaker, Konstanze Guggenberger, Nils Venhoff, Soroush Doostkam, Hans-Eckart Schaefer, Brita Fritsch

**Affiliations:** 1 Department of Neurology and Neuroscience, Medical Center, University of Freiburg, Breisacher Straße 64, D-79106 Freiburg, Germany; 2 Department of Neuroradiology, University of Freiburg, Breisacher Straße 64, D-79106 Freiburg, Germany; 3 Department of Rheumatology and Clinical Immunology, University of Freiburg, Breisacher Straße 64, D-79106 Freiburg, Germany; 4 Department of Neuropathology, University of Freiburg, Breisacher Straße 64, D-79106 Freiburg, Germany; 5 Department of Clinical Pathology, University of Freiburg, Breisacher Straße 64, D-79106 Freiburg, Germany

**Keywords:** Crohn’s disease, Cerebral vasculitis, Epilepsy, Central nervous system, Biopsy, Granulomatosis

## Abstract

**Background:**

Crohn’s disease (CD) is associated with a variety of extra-intestinal manifestations. Most commonly these involve the eye, skin, joints, coagulation system and liver. Cerebral manifestations of CD have been reported to a far lesser extent. The extensive detrimental impact of neurological symptoms on a patient’s quality of life makes an early diagnosis and treatment particularly important. In previous case-reports, diagnosis of cerebral manifestations in CD often relied upon magnetic resonance imaging (MRI) and computed tomography (CT) alone. To our knowledge, only one case-report has documented a histologically confirmed case of cerebral lesions associated with CD so far.

**Case presentation:**

A 39-year-old right-handed woman with a history of CD was referred to our hospital with etiologically unexplained Gadolinium (Gd)-enhancing cortical lesions, triggering epileptic seizures. A CT-scan of the thorax and bronchoalveolar lavage found no signs of sarcoidosis. Lumbar punctures and laboratory testing found no underlying infection or coincidental autoimmune disorders and MRI-scans showed progression of lesion load. Consequently, the patient underwent stereotactic biopsy of a cortical lesion. Histological examination revealed a mixed lympho-histiocytic and tuberculoid granulomatous inflammation surrounding small vessels and no signs for infection. After exclusion of other granulomatous diseases and the typical histological findings we diagnosed a cerebral granulomatosis as a manifestation of CD. The patient was initially started on azathioprine, which had to be switched to corticosteroids and methotrexate because of an azathioprine related pancreatitis. The patient has not suffered any further epileptic seizures to date.

**Conclusion:**

Cerebral manifestation of CD is a possibly underreported entity that may respond well to immunosuppressive treatment. In contrast to earlier reports of cerebral manifestations in CD, our patient showed no coincident gastrointestinal symptoms indicating an activity of CD during the progression of cortical lesion load, suggesting that similar to other extra-intestinal manifestations in CD, the activity of gastrointestinal symptoms does not necessarily reflect the activity of CD associated cerebral vasculitis. Therefore, diagnosis and therapy of cerebral manifestation may be delayed when focusing on gastrointestinal symptoms alone.

## Background

Crohn’s disease (CD) is an autoimmune disorder that primarily affects the gastrointestinal tract, but manifests itself in other organs as well [1]. The most common sites of extra-intestinal manifestation are joints, skin, eyes, coagulation system and liver. Cerebral manifestations of CD have been reported to a far lesser extent, contrasting the relatively high number of these reports in the context of other inflammatory bowel diseases [2–4]. Most commonly, cerebral manifestations in CD are a consequence of an affection of the coagulation system or a complication of medication [1, 5]. The extensive detrimental impact of neurological manifestations on a patient’s quality of life makes early diagnosis and treatment particularly important. In previous case-reports, diagnosis of cerebral manifestation in CD often relied upon MRI and CT alone [6–10]. To our knowledge, only one case-report has documented a histologically confirmed case of a cerebral manifestation in CD [11].

## Case presentation

This 39-year-old right-handed female suffered from epileptic seizures that began to develop in early 2014. The epileptic seizures were initiated by dyscognitive seizures and secondarily evolved into tonic-clonic seizures. The patient was started on anticonvulsive medication in August 2014 (Lamotrigin and Levetiracetam). Under this regimen, which was adapted regularly, the frequency of seizures stabilized at about one seizure every six months.

Some weeks prior to the first epileptic seizure, the patient had complained of novel subacute bifrontal headaches, which persisted intermittently over the course of the following months and were not directly correlated with the occurrence of epileptic seizures. Additionally, she reported a subjectively progressive impairment of her short-term memory. Her husband reported a temporary change in his wife’s personality during May 2015, which was accompanied by promiscuous behavior in social media and had not recurred since then.

Gastrointestinal symptoms (diarrhea, weight loss) had occurred for the first time in 2003. The diagnosis of CD was confirmed histologically via ileocolonoscopy in 2006. Symptoms were treated with a combination of steroids and mesalazine (5-ASA) from 2006 to 2008 and with methotrexate (15 mg once per week) from 2008 to 2016. The patient reported lack of gastrointestinal symptoms since 2013. Apart from nicotine abuse (25 pack-years) she reported no further comorbidities and did not suffer from any allergies. There was no family history of headaches, epilepsy, cancer and no exposure to toxic chemicals at home or at her workplace. There were no signs for lymphomatoid granulomatosis (no pulmonary and cutaneous lesions) or leukocyte oxidase deficiency (no recurrent infections). The patient did not travel outside of Europe in her entire life.

## Management and outcome

The first isolated dyscognitive seizure was interpreted as a transient ischemic attack (aphasia) in early 2014 and the patient was started on aspirin 100 mg daily after a cerebral hemorrhage was ruled out via computed tomography. In August 2014 the first secondarily generalized seizure led to the initiation of a standard MRI of the head, showing supratentorial Gd-enhancing cortical lesions that were deemed ischemic or inflammatory in nature. A lumbar puncture revealed an inflammatory constellation (14 cells/μl, negative oligoclonal bands). Bronchoalveolar lavage (including CD4/CD8-ratio) and a CT of the lungs were performed to test for (neuro-)sarcoidosis and provided normal results with no signs of perihilar lymphadenopathy. Furthermore, laboratory tests showed normal results for IL-2 receptor, ACE, neopterin, calcium, as well as for anti-neutrophil cytoplasmic antibodies (ANCA), anti-nuclear antibody (ANA) and anti-phospholipid-antibodies. An Interferon-gamma release assay test (QuantiFERON®) remained negative. A second MRI after two months (October 2014) showed further progression of the cortical lesions. Subsequently, the immunosuppressive therapy with methotrexate was complemented with steroids (initially 80 mg prednisolone, gradual reduction to 5 mg daily). A subsequent MRI in February 2015 showed a stable lesion load. Epileptic seizures in March, June and December 2015 as well as in March 2016 did not lead to adjustments of anticonvulsant or immunosuppressive therapy or to additional diagnostic measures.

**Fig. 1:**
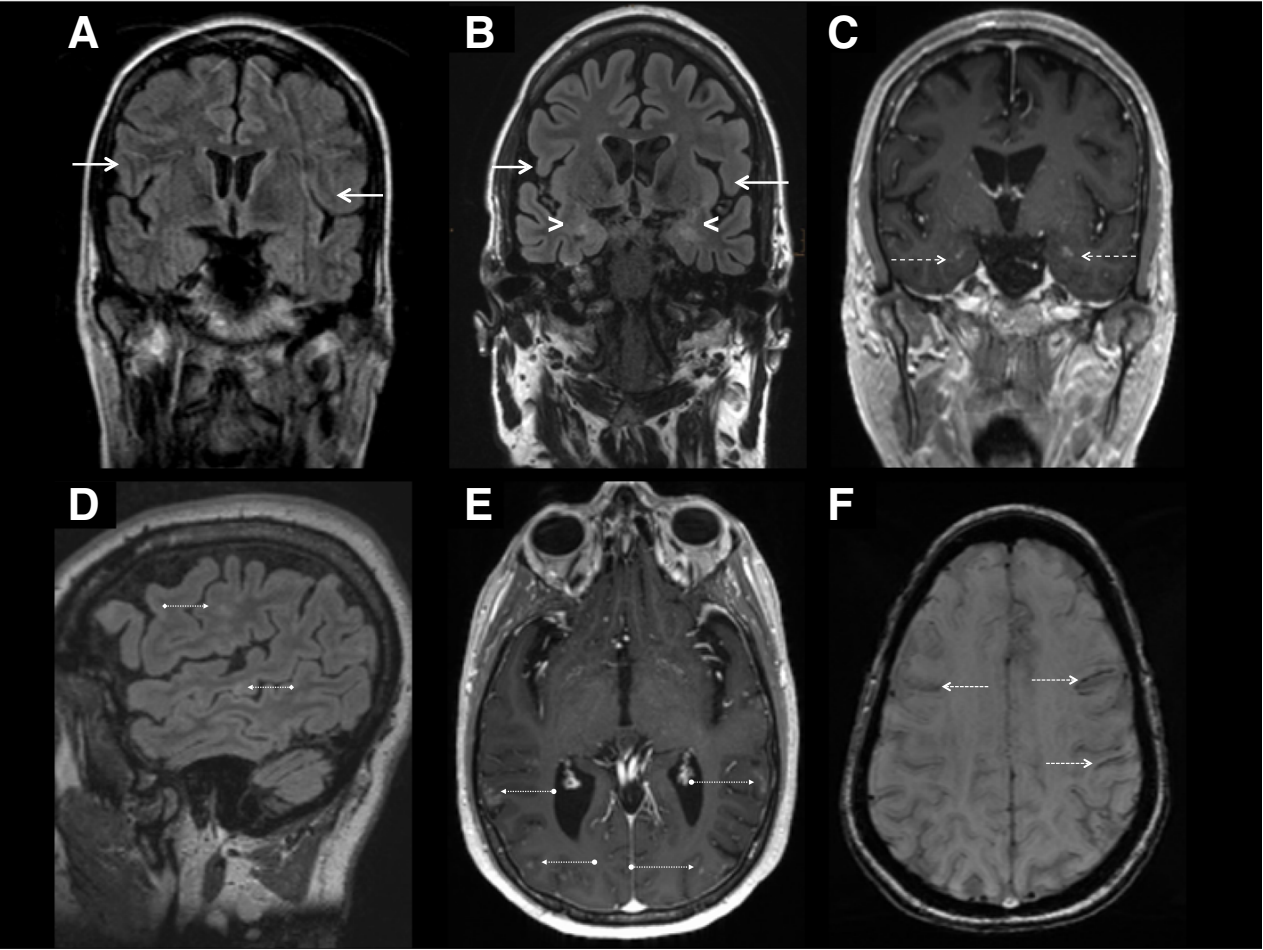
MRI-imaging reveals progressive atrophy of the temporal structures (**a**, **b**) and a new signal increase of the corpora amygdaloidea and hippocampi in FLAIR weighted sequences (**c**) over the course of time. Several spots of cortical hyperintensities are present in FLAIR weighted imaging (**d**). A faint contrast enhancement can be detected in the bihemsipheric cortex (**e**) and the corpora amygdaloidea (**c**). Mesencephalic and cortical hemorrhage (**f**) suggest an inflammatory process with microvascular vasculitis or an infectious or granulomatous genesis as possible differential diagnoses

In April 2016 the patient was referred to our university center for epilepsy. Here, the dosage of the anticonvulsive medication was increased and another MRI was initiated (August 2016), which showed an increase of lesions with numerous bihemispheric, hemorrhagically imbibed lesions of the cortex, basal ganglia, midbrain and pons. Gadolinium-enhancing lesions were revealed in both corpora amygdaloidea and cortically in both inferior frontal gyri, the left frontal medial gyrus, the left postcentral gyrus as well as in temporo-occipital and occipital regions in both hemispheres. Additionally, the MRI showed bilateral hippocampal atrophy and hyperintensity (Fig. 1). A comparative assessment between the MRI-scans was made difficult by their qualitative differences. A clear progression of lesion load could therefore only be established in comparison to the MRI-scans from 2014. Another lumbar puncture confirmed the previous pleocytosis (8 cells/μl, protein 508 mg/l) and now revealed positive oligoclonal bands in the cerebrospinal fluid (CSF). Antineural (Hu, Yo, Ri, CV2/CRMP5, amphiphysin, Ma1, Ma2, SOX1, GAD65, Tr(DNER), Zic4) and autoimmune encephalitis antibodies (VGKC (LGI1, CASPR2), GABA B, NMDA, AMPA1, AMPA2) remained negative in CSF and serum in two independent laboratories. Serum analysis of ANA, ANCA, phospholipid antibodies and rheumatoid factor remained negative as well. Complement factors were at normal levels. A neuropsychological test revealed depressive symptoms and potentially related impairments in attention, executive functions and long-term memory.

In September 2016 the patient was referred to our department of neurology via our colleagues at the university center for epilepsy in order to assess cerebral vasculitis. We performed another lumbar puncture (3 cells/μl, protein 584 mg/l) including antibody-tests for *borrelia burgdorferi* and *treponema pallidum*, polymerase chain reaction (PCR) tests for Herpes simplex viruses 1 and 2, Varicella-zoster virus, *tropheryma whipplei* and a HIV-test, which remained negative. An ultrasonography of the extra- and intracranial arteries of the neck and brain showed no signs of a vasculitis of the large vessels. The biomarkers for sarcoidosis (IL2-receptor, ACE, beta-2-microglobulin, lysozyme, neopterin) remained negative, as did proteinase3 antineutrophil cytoplasmic antibodies (PR3-ANCA), perinuclear anti-neutrophil cytoplasmic antibodies (p-ANCA) and blood cultures. There were no abnormalities in the differential blood count and in the immunoglobulins. A fluorescein angiography of the retina was performed in the department of Neuro-ophthalmology to test for a small-vessel vasculitis and Susac’s syndrome and revealed normal results.

**Fig. 2:**
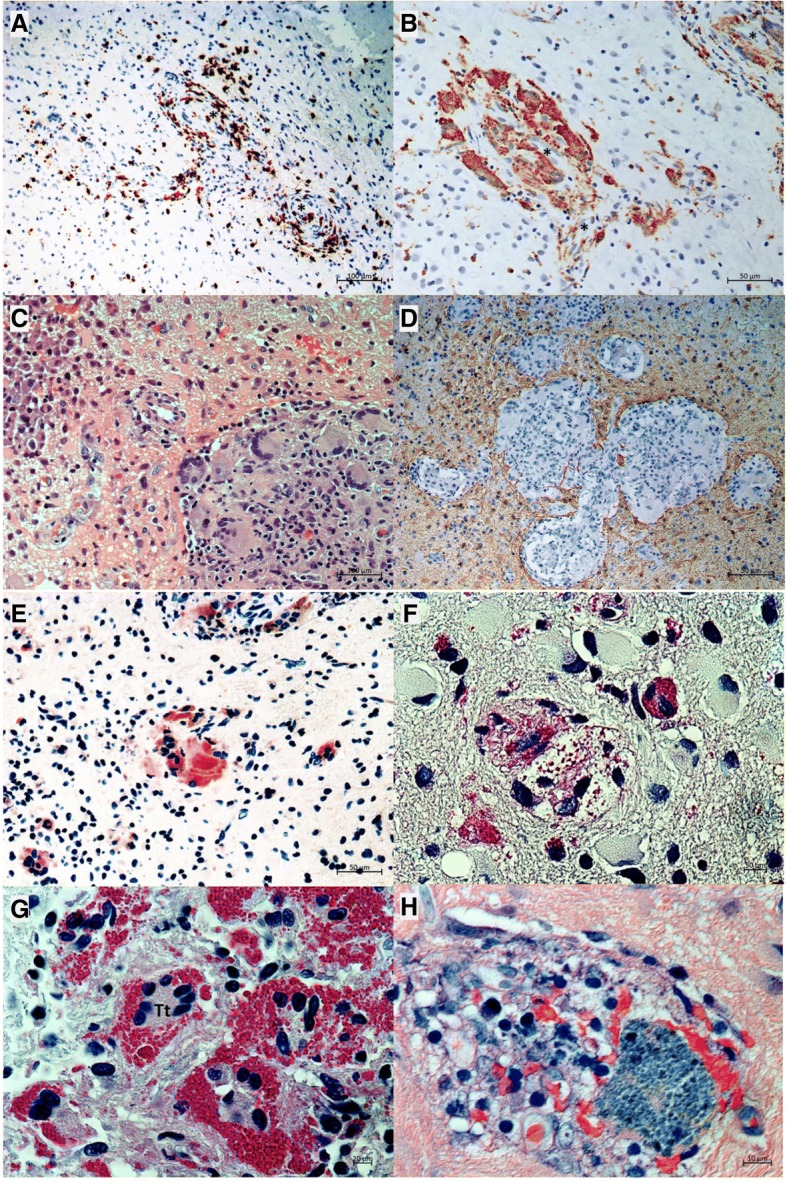
**a**, **b** Early granuloma formation, surrounding small blood vessels marked by asterics (*). **a** Immunohistochemical staining for CD3^+^ T-lymphocytes (brown staining). Serial sections (not depicted here) disclose an identical distribution of predominating CD4^+^ T-helper cells. **b** Serial section of the same vessel as shown in (**a**) at a higher magnification with CD68^+^ macrophages gathering at subintimal and adventitial spaces. **c**, **d** Full-blown granulomas. **c** Haematoxylin-Eosin staining displays a dense plasmocytic infiltrate at the left upper corner. The right lower corner encompasses a tuberculoid granuloma dominated by epithelioid cells and multinucleated Langhans-type giant cells. **d** A large granulomatous complex is embedded into sharply confined neuropil, highlighted by immunohistochemical staining for glial fibrillary acidic protein (brown colour). **e**-**h** Miscellaneous aspects of granuloma histiocytes. **e** Enzyme-histochemical staining for tartrate-resistant acid phosphatase (red colour) discloses a high activity of this particular isoenzyme of acid phosphatase, typically upregulated in mature macrophages involved in chronic lysosomal lipid degradation. **f** Periodic acid Schiff staining (PAS, red colour). A centrally placed foam cell contains translucent lipid vacuoles and few PAS^+^ ceroid-like granules. Additional smaller-sized macrophages are storing ceroid-like granules exclusively, whithout visible lipid vauoles. At the periphery of the graph, there are several gemistocytic astrocytes with large swollen PAS-negative cytoplasm. **g** Clustering PAS^+^ ceroid-storing macrophages fuse to multinucleated giant cells, partially of Touton-type (Tt). **h** On Giemsa-staining, some giant macrophages adopt a phenotype of sea-blue histiocytes

After completion of above-mentioned diagnostics, an interdisciplinary consent was reached to perform a stereotactic biopsy of one of the cortical lesions. Histology revealed leptomeningeal mixed mononuclear round cell infiltrates and multifocal mixed lympho-histiocytic and tuberculoid granulomatous lesions surrounding small vessels confirming the diagnosis of a cerebral granulomatosis (Fig. 2a-h). Histological, enzyme- and immunohistochemical examination showed no signs of infection with toxoplasmosis, mycobacteria or other bacteria or fungi as well as no signs for Amyloid-Beta Related Angiitis of the Central Nervous System (ABRA). There were no caseating and no geographical necroses.

On the basis of the histological analysis and negative tests for infectious causes we diagnosed a cerebral granulomatosis as a manifestation of Crohn’s disease in the central nervous system and began immunosuppressive treatment with azathioprine, which had to be switched to methotrexate and corticosteroids because of an azathioprine related pancreatitis. To date, the patient has not suffered any further epileptic seizures and revealed no signs of cognitive deterioration in the outpatient follow-ups.

## Discussion and conclusions

Cerebral manifestation of CD is a possibly underreported entity [5, 12, 13], which may respond well to immunosuppressive treatment [11]. In contrast to earlier reports of similar cases, our patient showed no clinical signs of an activity of CD during the progression of cortical lesion load. Similarly to other extra-intestinal manifestations in CD [14], the activity of gastrointestinal symptoms does not seem to be a reliable parameter in assessing the activity of cerebral granulomatosis. Therefore, diagnosis and therapy in CD may be delayed when the diagnostic focus lies on gastrointestinal symptoms alone. In our patient, time between symptom onset and diagnosis was two and a half years. To reduce progress of morbidity, an early stereotactic biopsy may be indicated in cases, in which MRI-scans are not sufficient for confidently assessing the etiology of progressive cerebral lesions.

## Abbreviations

5-ASA: Mesalazine
ABRA: Amyloid-Beta Related Angiitis of the Central Nervous System
ACE: Angiotensin-converting enzyme
ANA: Anti-nuclear antibody
ANCA: Anti-neutrophil cytoplasmic antibody
CD: Crohn’s disease
CSF: Cerebrospinal fluid
CT: Computed tomography
CT: Computed tomography
Gd: Gadolinium
HIV: Human immunodeficiency virus
MRI: Magnetic resonance imaging
p-ANCA: Perinuclear Anti-Neutrophil Cytoplasmic Antibodies
PCR: Polymerase chain reaction
PR3-ANCA: Proteinase-3 antineutrophil cytoplasmic antibodies
